# Impact of Nursing Professional Values on Depression, Stress, and Anxiety among Nurses during the COVID-19 Pandemic

**DOI:** 10.1155/2024/5199508

**Published:** 2024-08-08

**Authors:** Alberto Lana, Beatriz Sánchez-García, María González-García, Ana Fernández-Feito, David González-Pando

**Affiliations:** ^1^ Department of Medicine University of Oviedo, Avda Julián Clavería s.n, Oviedo 33006, Spain; ^2^ Healthcare Research Area Health Research Institute of Asturias (ISPA), Av. Roma, s.n, Oviedo 33011, Spain; ^3^ School of Nursing of Gijón University of Oviedo, Camino de los Prados, 394, Gijón 33203, Spain

## Abstract

**Aim:**

The aim was to explore the association between nursing professional values (NPV) and mental health among registered nurses (RN) in Spain.

**Background:**

Nursing is a profession rooted in strong professional values, which guide and shape clinical practice and occupational behaviors. NPV should serve as a source of support in situations of great uncertainty.

**Methods:**

A cross-sectional study was conducted during the remission phase of the second wave of the COVID-19 pandemic (December 2020-January 2021) among a sample of Spanish RN (*n* = 420). NPV were assessed using the Nursing Professional Values Scale (NPVS-R), comprising 26 items grouped into five factors: caring, activism, trust, professionalism, and justice. Perceived stress, anxiety, and depression were measured by the Perceived Stress Scale (PSS-14) and the Hospital Anxiety and Depression Scale (HADS). Adjusted linear regressions were used to estimate b coefficients for the associations between NPV scores and the three mental health indicators.

**Results:**

The fully-adjusted analysis, including sociodemographic and occupational variables, revealed that higher activism scores were associated with higher scores of stress (b coefficient: 0.46; 95% confidence interval: 0.03–0.88; *p* value: 0.035), anxiety (0.24; 0.05–0.43; 0.014), and depression (0.19; 0.01–0.36; 0.035). No other NPV was associated with mental health.

**Conclusion:**

Organizational policies and programs should be established to protect the most activist RNs and to mitigate the potential detrimental effect of activism on mental health at times and/or circumstances of high workloads and personal stress.

## 1. Introduction

Nurses are one of the occupational groups that present the highest risk of suffering from mental health distress. Contact with illness and suffering, together with working under the clinical uncertainty of most processes, are inexhaustible sources of psychological stressors, emerging from the nature of care itself. The most prevalent mental health issues suffered by nurses are compassion fatigue and burnout [1, 2], which are in turn associated with anxiety and depression [3, 4]. A meta-analysis of 79 studies involving 28,509 nurses from 11 countries found moderate levels of compassion fatigue, which has increased gradually worldwide over the last decades [2]. Another meta-analysis of 61 studies involving 45,539 nurses from 49 countries found an overall pooled prevalence of burnout of 11.2% among nurses globally [5].

In addition, the physical and psychological stress of providing care in complex occupational environments means that nurses are even more vulnerable to mental health problems. Certain situations and characteristics of the work environment may reduce the mental well-being of nurses, which compromises the quality of care [6]. Work overload, work shifts, high staff turnover, difficulty in reconciling work and private life, or the type of unit are some of the most evident contributing factors [7]. These contributors can trigger physical exhaustion, sleep disorders, headache, osteoarticular pain, difficulty concentrating, and memory losses, among other symptoms, resulting in job withdrawal due to sick leave, absenteeism, and intention to leave [5, 8]. Furthermore, other less-visible characteristics of the nursing profession confer nurses with a greater propensity for mental distress. First, the mismatch between nurses' expectations and the nonideal reality of nursing care (i.e., conflict between vocation and role) [9]. Second, the stereotypes and gender roles around nursing profession, which can prevent the recognition of mental fatigue from caregiving [10].

These circumstances converged and became intensified during the first waves of the global COVID-19 pandemic, with a severe impact on the mental health of nurses around the world. Several literature reviews have provided evidence of the negative impact of the COVID-19 pandemic on relevant indicators of nurses' mental health, such as mental overload, insomnia, anxiety, depression, post-traumatic stress syndrome, and other mental health disorders [11–15]. Other devastating legacies of the COVID-19 pandemic were the overflowing waiting lists that tested the patience and health of users, and the emerging mistrust of the scientific establishment and the healthcare system by large segments of the population, thus increasing the burden on healthcare workers. Conversely, some studies conducted among healthcare students found a positive impact of the COVID-19 pandemic on professional values [16, 17]. Thus, the COVID-19 pandemic provided a unique setting to study the role of certain professional characteristics on nurses' mental well-being [18, 19].

**Figure 1:**
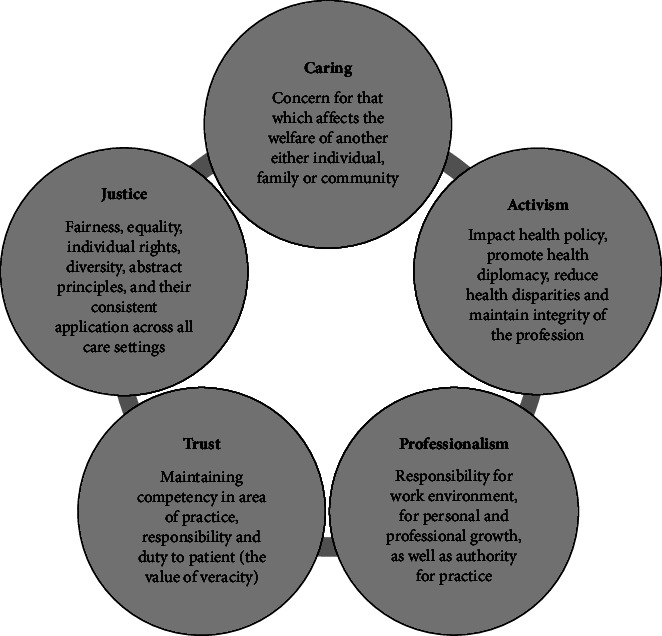
Core professional values of nursing according to Weis and Schank [21, 25, 26].

Nursing professional values (NPV) are the principles that govern the nursing discipline which have been agreed upon by different nursing associations worldwide. Professional values provide a nursing moral framework and shape ethical behavior, thereby aiding in complex decision making and maintaining nurses' moral obligation to follow organizational standards and not violate ethical principles [20]. Weis and Schank [21] defined professional values as “standards of action accepted by professionals and professional groups which provide a framework for evaluating beliefs and attitudes that influence behavior”. Therefore, in daily professional practice, they provide the basis for care decisions [22, 23]. Although NPVs originate from each region's nursing history, cultural background, social groups, religion, and lived experiences, according to the American Nurses Association (ANA) [24], certain NPVs represent the ethical code of the profession and can be considered universal: altruism, autonomy, human dignity, social justice, and integrity [25]. A conceptual model for NPVs derived from the work of Weis and Schank [21, 25–27], which in turn is based on the ANA Code of Ethics, is shown in Figure 1.

Nurses' awareness of their professional values and how they impact their caring role constitute a central part of humanistic nursing care [28]. Ultimately, strong NPVs should contribute to adequate and safe care [20], even in complex clinical or organizational situations, which increase the physical and psychological demands on nurses. According to the results of a cross-sectional study in Spain, NPVs were positively associated with compassion satisfaction during the COVID-19 pandemic [29]. We therefore hypothesized that NPVs would allow nurses to manage their levels of stress, anxiety, and depression under highly stressful conditions and work overload. This study aimed to explore the association between NPVs and mental health indicators of registered nurses (RNs) in Spain during the COVID-19 pandemic, specifically analyzing stress, anxiety, and depression.

## 2. Methods

### 2.1. Study Design and Participants

A cross-sectional study involving a sample of RNs of Asturias, a region in northern Spain of approximately 1 million inhabitants, with a network of 15 hospitals and 60 primary care centers. A minimum required sample size of 345 nurses was deemed necessary to detect clinically relevant differences in mental health indicators, considering a 3% margin of error and a 95% confidence interval. Participants were recruited through the Official Board of Nurses of Asturias using nonprobabilistic sampling. This institution integrates all the RNs who carry out their professional practice in the region, since professional membership is mandatory in Spain. An e-mail was sent to all the RNs (*N* = 4,550), providing detailed information on the study and a link to an anonymous online form containing the study survey. In addition, participants were asked to provide informed consent and confirm that they met the established eligibility criteria when accessing this online form. RNs were included if they worked in any public or private health facility in Asturias and if they had patient contact since the beginning of the COVID-19 pandemic (March 2020). Conversely, RNs on sick leave due to accident, mental or physical illness between March 2020 and January 2021 were excluded. To maximize recruitment, information about the study and the link to the questionnaire were also posted on the institutional website of the Official Board of Nursing of Asturias.

Data collection was performed between December 2020 and January 2021, during the remission phase of the second wave of the pandemic in Asturias, the most severe in terms of cases and deaths of the entire COVID-19 pandemic. In November 2020, dubbed Black November, Asturias recorded the highest 14-day notification rate of the COVID-19 pandemic, with 650 cases per 100,000 inhabitants, which was well above the Spanish average. This month alone accounted for 46% of all COVID-19 deaths in 2020.

Prior to the mass mailing, a pilot test of the procedure and the survey were performed with 40 randomly selected RNs. By the end of the recruitment process, 435 surveys were received. Later, six duplicate records and nine participants with missing data on occupational variables were removed. Therefore, the final sample consisted of 420 RNs, representing 9.3% of the target population. Further details about the study are reported elsewhere [29].

### 2.2. Ethical Considerations

The study was approved by the Research Ethics Committee of Asturias (ref. 563/2020) and all participants gave informed consent. Participation was voluntary and anonymous. No incentives were offered. Data confidentiality and patient anonymity were preserved in compliance with the Spanish Organic Law 3/2018 of 5 December on Personal Data Protection and Guarantee of Digital Rights. This manuscript follows the Strengthening the Reporting of Observational Studies in Epidemiology (STROBE) recommendations.

### 2.3. Study Variables and Survey Instruments

#### 2.3.1. Nursing Professional Values

NPVs were measured using a reduced version of the Nursing Professional Values Scale (NPVS-R) [26, 30], an instrument derived from the Code of Ethics of the American Nurses Association. The NPVS-R consists of 26 items grouped into five dimensions: caring (9 items), activism (5 items), trust (5 items), professionalism (4 items), and justice (3 items). The importance assigned to each value is scored according to a Likert-type scale of 1 to 5 points, with 1 being “not important” and 5 being “very important.” Weis and Schank supported the internal consistency reliability of the five factors (alpha coefficients of 0.70 to 0.85) and of the total scale (alpha coefficient of 0.92) [26].

#### 2.3.2. Mental Health

In our study, the mental health of the RNs was assessed by considering the self-perceived level of stress, and symptoms of anxiety and depression. Stress was measured using the Spanish version of the Perceived Stress Scale (PSS-14) [31]. With this tool, each RN assessed the degree of stress that certain day-to-day situations caused them during the month prior to the response. The PSS-14 consists of 14 items with five response options, consisting of 0 “never,” 1 “almost never,” 2 “from time to time,” 3 “often,” and 4 “very often,” although the score of some items must be reversed. The total score ranges from 0 to 56 points, with higher scores indicating higher levels of perceived stress. Cronbach's alpha coefficient for this study was calculated to be 0.90. Consequently, it was determined that the scale had a high level of reliability for our sample.

Anxiety and depression were assessed using the Spanish version of the Hospital Anxiety and Depression Scale (HADS). This scale consists of two independent subscales of seven items each, which assess anxiety and depression symptoms during the previous seven days [32, 33]. Each item has four response options, ranging from 0 “absence/minimal presence” to 3 “maximum presence.” Some items must be reversed to obtain the final HADS score. The score for each subscale ranges from 0 to 21 points, with a higher score indicating greater severity of symptoms. In our sample, we obtained an alpha of 0.92, which is considered an excellent reliability.

#### 2.3.3. Potential Confounders

A set of potential confounding variables was generated based on a literature review followed by expert consensus. Sociodemographic data collected in the study included sex (male or female), age (in years), sentimental partner (yes or no), and having children (yes or no). Regarding work variables, the level of education (university degree or postgraduate degree, including master's or doctorate), type of service (COVID-19 frontline unit, including emergency department, intensive care units and COVID-19 units, or second-line unit, including other services) and work shift (fixed morning, rotating sift or morning on-call) were considered.

### 2.4. Data Analysis

Statistical analyses were performed using STATA version 19 (StataCorp.; College Station, Texas). First, a Shapiro–Wilk normality test was performed on the data set of each measure and the results indicated that scores followed a normal distribution. Subsequently, linear regressions were used to obtain the b coefficients of the associations between the NPV scores and the three mental health indicators. Positive b coefficients indicated a direct association and negative coefficients indicated an inverse association. To rule out the potential confounding effect of some variables on the associations studied, in addition to a crude model, regression models were run adjusting for sociodemographic variables (age, sex, relationship, and children) and work variables (academic level, unit, and shift). Given that there was a strong correlation between all the NPVs (Table 1), each model was adjusted for the remaining NPVs. For example, when the NPV care was used as an independent variable, the model was additionally adjusted for activism, trust, professionalism, and justice. In this manner, the isolated contribution of each NPV on mental health was studied. Only *p* values <0.05 were considered statistically significant.

## 3. Results

The social and work characteristics of the sample are shown in Table 2. Most participants were women, aged 35–50 years and without children. Regarding the occupational variables, the most frequent were having completed only a university degree in nursing, second-line positions during the COVID-19 pandemic, and rotating work shifts.

The mean scores (± standard deviation) for the five dimensions of the NPVs were: 41.1 points for caring, 20.4 for activism, 22.4 for trust, 17.1 for professionalism, and 13.5 for justice (Table 3). Regarding the selected mental health indicators, the mean score for stress was 27.5 (±9.71), 8.91 (±4.39) for anxiety, and 6.66 (±4.01) for depression.

According to the results of the linear regressions for the association between PNVs and mental health indicators, only activism was significantly and directly associated with stress, anxiety, and depression (Table 4). The higher the activism score, the lower the mental well-being score of the RNs, both in the crude models and those adjusted for confounding variables.

## 4. Discussion

According to the results of our study, conducted in the context of a situation of maximum clinical and ethical uncertainty, organizational difficulties, and high physical and emotional demands on healthcare workers, the activism of RNs was associated with worse mental health indicators. No other NPV was associated with the mental well-being of RNs.

Our study was the first to examine the association between NPV and multiple mental health indicators, hampering comparison with other studies. Overall, our findings did not support the study hypothesis, as NPVs did not mitigate the negative impact of the pandemic on the mental health of RNs. A plausible explanation is that the mental health of RNs was so affected by the demands of the COVID-19 pandemic that the ability of NPVs to guide clinical practice while maintaining mental well-being was severely reduced. Moreover, contrary to the hypothesis, higher activism scores were consistently associated with lower levels of stress, anxiety, and depression. Activism refers to an active role of RNs in aspects related to resource management, research, and transfer of relevant findings to clinical practice. It also represents the struggle to strengthen the profession and broaden its scope of action, with active participation in professional organizations. In addition, activism is considered necessary for leadership. Therefore, it refers to a conception of the profession that, without neglecting individual-centered care, is oriented towards the global health of populations, and seeks to empower the nursing discipline by participating in health policies [27]. In short, activism is inherent to the nursing profession, since RNs are the advocates and interlocutors of the people in relation to the healthcare system [34]. Although it is not one of the dominant NPVs in normal situations [35], its characteristics may make activism particularly relevant in exceptional socio-health situations that require RNs to take a step forward.

The association between activism and poorer mental health has already been documented by some other authors, who found that activism was related to lower job satisfaction and higher emotional burden and could increase stress and anxiety [36–38]. The reasons that may explain this association are varied. First, the mental and emotional sacrifice demanded by activism may contribute to the onset of anxiety and depressive symptoms [36]. Getting involved in organizational issues and striving to translate recent findings into clinical practice can be mentally challenging and emotionally draining, because it usually pits the activist against management, peers, and one's own personal limitations. For this reason, building a positive view of activism would help mitigate this deleterious effect. Mundie et al. [39] emphasize the importance of creating support networks for activist RNs as a way to ensure quality, sustainable, and protective nursing care for workers. Second, because activism is time-consuming, highly activist RNs need to put more effort into direct patient care to meet their expectations, which may contribute to a sense of lack of accomplishment, leading to increased stress and anxiety. Third, activism may be associated with exposure to situations of ethical conflict. Nurses who engage in activism-related initiatives often do so to achieve improvements in patients' rights or to defend the healthcare system, resulting in situations where ethics clash with the requirements imposed by institutional policies. This is especially relevant in exceptional situations and can trigger moral distress, sadness, and disillusionment at not being able to act according to their value system [40]. When there is a confrontation of the activist RN with the organization or the system, feelings of isolation and stigmatization may emerge, especially if the activist behavior is seen as a threat by management or peers [41].

Of the remaining NPVs included in this study, the only one that showed an association with a significant borderline *p* value was professionalism. In the crude analyses and adjusted for sociodemographic variables, a higher score in professionalism was associated with a lower level of stress. Kim et al. [42] observed a positive link between professionalism and quality of life in RNs, which can be considered a proxy for good mental health. Furthermore, in this study, professionalism was also associated with a better ethical climate and higher compassion satisfaction. In contrast, Gonzalez-Pando et al. have suggested that higher levels of expertise, which indicate active engagement in professionalism, may increase compassion fatigue among frontline nurses [29].

Clearly, all countries must learn to live with health crises, thus, it is imperative to build robust and resilient health systems that also protect their workers through positive work prospects and safe and healthy work environments [18, 19]. Although in this study NPVs were not associated with greater mental well-being, as hypothesized, preserving NPVs should be non-negotiable. A healthcare system that cultivates NPVs will always strive for excellence and safety and will place its staff and users at the center of care. Therefore, although our findings might discourage the promotion of activism, it should still be considered a core value, which strongly supports the precepts of nursing codes of ethics around the world. Indeed, one of the main factors explaining greater activism is having received a higher quality university education [43]. Rather than attempting to improve the mental health of RNs by curtailing their activism, which would undermine the social and health outcomes of the nursing profession, it would be advisable to have programs to encourage activism in a rational manner and simultaneously design programs for the mental healthcare of the workforce. In addition, a cross-sectional study of 748 nurses from Saudi Arabia found that activism during the COVID-19 pandemic was significantly associated with higher professional competence [44], which may be another compelling reason to promote continuing education programs that emphasize the importance of NPVs.

In general, close monitoring of the psychological status of nurses and its determinants should be part of the ongoing preparedness efforts of health systems worldwide [45]. In addition, there is an urgent need to develop and implement local and national strategies, consistent with NPVs, to help nurses cope with the burden of mental and psychological disorders resulting from the demanding and emotionally draining work of caring for people and communities [46]. With the prospect of emerging health crises, this is an even higher priority.

This study had some limitations. First, the study design was cross-sectional; therefore, it is not possible to know the precise direction of the associations. However, considering the nature of the variables studied and previous scientific literature, the direction proposed in this study is plausible and more likely than the opposite. Second, because of the convenience sampling, the representativeness of the sample cannot be guaranteed. Although the number of responses was sufficient to detect relevant changes, selection bias cannot be ruled out. Respondents to voluntary web-based forms tend to be more interested in the topic of study and younger, which may affect the results. As the survey was delivered at times of high professional and personal demand due to the COVID-19 pandemic, it is possible that RNs with worse mental health indicators, more upset with the organization, and those who are more militant may have responded, which may have affected our findings. Third, although the associations found regarding activism were statistically significant and consistent across models, they were clinically irrelevant. Finally, some variables of interest for a better understanding of the phenomenon, such as personality factors, were not included; their measurement would have greatly complicated data collection.

## 5. Conclusion

In conclusion, higher activism scores were associated with worse mental health indicators in Spanish RNs, including higher levels of stress, and symptoms of anxiety and depression. No other NPV was consistently associated with mental health. Organizational policies and programs should be established to protect the most activist RNs and to mitigate the potential detrimental effect of activism on mental health at times and/or circumstances of high work and personal demand. Well-designed studies are needed to test the longitudinal association between professional values and the mental health of RNs, as well as that of other healthcare workers.

## Figures and Tables

**Table 1:** Correlation between nursing professional value scores (*n* = 420).

	Care	Activism	Trust	Professionalism	Justice
Care	—	—	—	—	—
Activism	0.59^*∗∗∗*^	—	—	—	—
Trust	0.71^*∗∗∗*^	0.63^*∗∗∗*^	—	—	—
Professionalism	0.58^*∗∗∗*^	0.77^*∗∗∗*^	0.70^*∗∗∗*^	—	—
Justice	0.71^*∗∗∗*^	0.69^*∗∗∗*^	0.74^*∗∗∗*^	0.63^*∗∗∗*^	—

Values are Pearson's correlation coefficients. ^*∗∗∗*^*p* < 0.001.

**Table 2:** Characteristics of the sample (*n* = 420).

	Participants, *n* (%)	NPV, mean score (sd)				
		Care	Activism	Trust	Professionalism	Justice
Sex						
Male	55 (13.1)	41.3 (4.07)	20.1 (4.33)	22.4 (2.58)	17.0 (3.15)	13.4 (1.83)
Female	365 (86.9)	41.1 (4.50)	20.5 (3.70)	22.4 (2.53)	17.1 (2.94)	13.5 (1.83)
*p* value^a^		0.765	0.456	0.784	0.840	0.715
Age						
<35 years	133 (31.7)	40.7 (4.97)	20.2 (3.92)	22.3 (2.62)	16.8 (3.07)	13.3 (1.88)
35–50 years	178 (42.4)	41.4 (4.94)	20.7 (3.72)	22.5 (2.40)	17.4 (2.76)	13.6 (1.75)
>50 years	109 (26.0)	41.1 (4.12)	20.3 (3.75)	22.4 (2.64)	16.9 (3.13)	13.5 (1.89)
*p* value^a^		0.357	0.367	0.786	0.164	0.365
Relationship						
No partner	90 (21.4)	41.1 (4.37)	20.0 (4.06)	22.5 (2.48)	17.1 (2.88)	13.5 (1.83)
In a relationship	330 (78.6)	41.1 (4.71)	20.5 (3.71)	22.3 (2.71)	16.7 (3.25)	13.6 (1.86)
*p* value^a^		0.960	0.203	0.564	0.242	0.660
Children						
No children	235 (56.0)	40.9 (4.66)	20.1 (3.95)	22.4 (2.59)	16.8 (3.09)	13.5 (1.85)
Children	185 (44.0)	41.4 (4.15)	20.9 (3.54)	22.5 (2.46)	17.3 (2.78)	13.6 (1.81)
*p* value^a^		0.352	0.051	0.689	0.087	0.627
Educational level						
Degree	327 (77.9)	41.1 (4.43)	20.3 (3.86)	22.4 (2.59)	17.0 (2.98)	13.5 (1.82)
Postgraduate degree	93 (22.1)	41.2 (4.51)	20.9 (3.51)	22.6 (2.30)	17.2 (2.89)	13.5 (1.87)
*p* value^a^		0.762	0.136	0.502	0.534	0.834
Type of service						
Frontline unit	156 (37.1)	41.0 (4.26)	20.1 (4.04)	22.4 (2.48)	16.7 (3.10)	13.3 (2.02)
Second line unit	264 (62.9)	41.2 (4.55)	20.6 (3.66)	22.4 (2.57)	17.2 (2.88)	13.6 (1.70)
*p* value^a^		0.745	0.151	0.751	0.293	0.131
Work sift						
Fixed morning	111 (26.4)	41.2 (4.38)	20.4 (3.86)	22.3 (2.59)	17.1 (3.01)	13.6 (1.68)
Rotating shift	228 (54.3)	41.0 (4.45)	20.4 (3.67)	22.4 (2.49)	17.0 (2.87)	13.4 (1.87)
Morning shift + on-call	81 (19.3)	41.4 (4.51)	20.5 (4.06)	22.6 (2.58)	17.1 (3.19)	13.6 (1.92)
*p* value^a^		0.658	0.985	0.749	0.986	0.799

NPV: nursing professional value, ^a^Unpaired *t*-tests and one-way ANOVA tests were used to compare the means of each NPV score by selected variables.

**Table 3:** Nursing professional value scores (*n* = 420).

	Theoretical range	Observed range	Mean (SD)
Care	9–45	16–45	41.1 (4.44)
Activism	5–25	6–25	20.4 (3.79)
Trust	5–25	12–25	22.4 (2.53)
Professionalism	4–20	5–20	17.1 (2.96)
Justice	3–15	5–15	13.5 (1.83)

**Table 4:** B coefficients (95% CI) for the association between professional nursing values scores and mental health indicators (*n* = 420).

	Stress		Anxiety		Depression	
	Coef. B (95% CI)	*p* value	Coef. B (95% CI)	*p* value	Coef. B (95% CI)	*p* value
Care						
Crude model	−0.18 (−0.50; 0.15)	0.280	−0.01 (−0.16; 0.13)	0.848	−0.07 (−0.20; 0.07)	0.323
Adjusted model^a^	−0.17 (−0.50; 0.15)	0.303	−0.02 (−0.17; 0.12)	0.758	−0.07 (−0.21; 0.06)	0.294
Activism						
Crude model	0.45 (0.27; 0.87)	0.037^b^	0.24 (0.05; 0.43)	0.014^b^	0.18 (0.01; 0.35)	0.042^b^
Adjusted model^a^	0.46 (0.03; 0.88)	0.035^b^	0.24 (0.05; 0.43)	0.014^b^	0.19 (0.01; 0.36)	0.037^b^
Trust						
Crude model	0.15 (−0.49; 0.80)	0.641	−0.02 (−0.31; 0.28)	0.915	0.03 (−0.23; 0.30)	0.807
Adjusted model^a^	0.12 (−0.52; 0.77)	0.710	−0.03 (−0.33; 0.26)	0.836	0.03 (−0.24; 0.30)	0.807
Professionalism						
Crude model	−0.58 (−1.12; −0.04)	0.036	−0.22 (−0.46; 0.03)	0.084	−0.18 (−0.41; 0.04)	0.114
Adjusted model^a^	−0.54 (−1.08; 0.00)	0.050	−0.21 (−0.45; 0.04)	0.102	−0.17 (−0.40; 0.05)	0.130
Justice						
Crude model	−0.31 (−1.18; 0.56)	0.489	−0.16 (−0.56; 0.23)	0.426	−0.18 (−0.54; 0.18)	0.322
Adjusted model^a^	−0.32 (−1.20; 0.55)	0.467	−0.13 (−0.53; 0.27)	0.520	−0.18 (−0.55; 0.18)	0.323

^a^Model adjusted for sex, age, relationship, children, educational level, type of service, and work shift. ^b^Statistically significant association.

## Data Availability

The data used to support the findings of this study are available from the corresponding author upon reasonable request.
